# Exposure to workplace bullying: the incremental effect of gelotophobia beyond the big five

**DOI:** 10.3389/fpsyg.2024.1400940

**Published:** 2024-04-25

**Authors:** Filip Sulejmanov, Kryštof Petr, Jana Gambová, Tracey Platt, Martin Seitl

**Affiliations:** ^1^Department of Psychology, Palacký University Olomouc, Olomouc, Czechia; ^2^School of Psychology, University of Sunderland, Sunderland, United Kingdom

**Keywords:** workplace bullying, NAQ-R, big five, gelotophobia, incremental validity

## Abstract

The role of Big Five personality traits in exposure to workplace bullying has been a focus of numerous studies. Yet less is known about the incremental validity of narrower personality constructs. The aim of the present study was to investigate the incremental effect of gelotophobia (the fear of being laughed at) in predicting exposure to workplace bullying beyond the Big Five personality domains. The sample comprised 328 employees (77% females) from different regions of the Czech Republic. Correlational analysis showed that negative emotionality and gelotophobia were related to workplace bullying in theoretically expected ways. Results from a multiple regression indicated that gelotophobia had an incremental effect in predicting exposure to workplace bullying over and above the personality domains. Overall, this study provides new insights and extends previous investigations concerning the role of gelotophobia in workplace bullying. We also discuss the limitations of our study and provide suggestions for future research.

## Introduction

Two main perspectives, namely the work environment hypothesis and the individual dispositions hypothesis, are typically used to explain the antecedents of workplace bullying [Nielsen and Knardahl, 2015; also see Leymann (1996), Zapf and Einarsen (2001), and Balducci et al. (2021)]. From the work environment perspective, factors such as job design, organizational climate and culture, and leadership are usually seen as crucial for the occurrence of workplace bullying (Gamian-Wilk et al., 2022), while on the other hand, the individual dispositions hypothesis (also known as the vulnerability hypothesis) focuses on individual characteristics which might increase the risk of being a target or a victim of bullying [Nielsen and Knardahl, 2015, see also Gamian-Wilk et al. (2022)].

In the present study, we follow the vulnerability hypothesis – keeping in mind that the perspectives should be seen as complementary, but not opposite – and investigate the role of the Big Five domains (Soto and John, 2017a) and the incremental validity of gelotophobia (Ruch and Proyer, 2008a) in predicting workplace bullying, understood as “the persistent exposure to interpersonal aggression and mistreatment from colleagues, superiors or subordinates” (Einarsen et al., 2009, p. 24). In particular, we consider workplace bullying as operationalized with the Negative Acts Questionnaire-Revised (NAQ-R) (Einarsen et al., 2009), which includes three different forms of exposure to bullying (work-related, person-related, and physically intimidating). Moreover, we introduce a dimension labeled as humor-related bullying. The role of the personality dimensions and gelotophobia in predicting self-labeled victimization from bullying is one of the interests of this study as well.

### Personality and workplace bullying

In a meta-analysis, investigating the relationship between the Five-Factor Model of personality (McCrae and Costa, 1987) and exposure to harassment (a higher-ordered construct including different forms of psychological mistreatment), Nielsen et al. (2017) reported that being exposed to harassment was positively associated with neuroticism, and negatively related to extraversion, agreeableness, and conscientiousness. The theoretical relations between the personality dimensions and harassment were built upon the four mechanisms explaining the relationship between bullying and individual dispositions (Nielsen and Knardahl, 2015).

In sum, Nielsen and Knardahl (2015) propose that bullying and individual dispositions relationship can be explained by (1) the no-relationship mechanism; indicating no association, (2) the target-behavior mechanism; implying that employees with certain dispositions not only fail to meet expectations but also irritate others, possibly by violating the usual norms of polite and friendly interaction, which can lead to others responding with aggressive behaviors, (3) the negative perceptions mechanism; suggesting that specific individual dispositions are related with a lowered threshold for interpreting behaviors as harassing, and therefore, employees with such dispositions have a higher risk than others for labeling negative events at the workplace as bullying, and (4) the reverse causality mechanism; individual dispositions are viewed as outcomes rather than precursors of workplace bullying.

Nielsen et al. (2017) highlight that the findings regarding the association between bullying and personality should not be used to conclude whether dispositional characteristics among those harassed are causes or consequences of harassment, as their study was cross-sectional [see Bowling et al. (2010), Nielsen and Knardahl (2015), and Podsiadly and Gamian-Wilk (2017) for longitudinal research findings]. As our study is also cross-sectional in nature, we firstly focus on replicating the above findings in a Czech cultural setting. Moreover, we utilize a more recent operationalization of the Big Five dimensions [see Soto and John (2017a,b)]. Soto and John (2017a) used the following labels for the Big Five personality dimensions: Extraversion, Agreeableness, Conscientiousness, Negative Emotionality (also known as Neuroticism; see, e.g., McCrae and Costa, 2008), and Open-Mindedness [also known as Openness to Experience, Intellect, or Imagination; see Goldberg (1993), John et al. (2008), and McCrae and Costa (2008)]. The Big Five Inventory-2 (BFI-2) operationalizes these domains using a total of 60 items, and it was shown that it has a robust hierarchical structure, controls for individual differences in acquiescence, and has conceptual breadth, specificity, and predictive power (Soto and John, 2017a). In the present study, we employ the abbreviated 30 items version of the scale, i.e., the BFI-2-S (Soto and John, 2017b).

### The role of gelotophobia

Gelotophobia is defined as the pathological fear of being an object of laughter or appearing to others as a ridiculous object (Ruch and Proyer, 2008b; Titze, 2009). Individuals scoring high on gelotophobia do not perceive humor and laughter as relaxing and joyful social experiences (Titze, 2009) and they fail to discriminate between ridicule and good-humored teasing (Platt, 2008). Clinically observed behaviors described by Titze (1997, 2009) was developed into a model of the causes and consequences of gelotophobia (Ruch and Proyer, 2008a), which later was updated to include putative causes and the moderating factors (Ruch et al., 2014). Ruch and Stahlmann (2020) advanced the framework to a dynamic model of gelotophobia, providing an updated definition that is anchored in the construct of vulnerability. Gelotophobia is understood “as a distinguishable pattern of lacking resources (i.e., misinterpretation of joy and laughter) that can result in negative consequences (e.g., reduced well-being and performance) if individuals have no access to further resources (e.g., social support) or are exposed to severe stressors (e.g., workplace bullying)” (Ruch and Stahlmann, 2020, p. 16,369).

By far, a few studies have investigated the association between gelotophobia and (workplace) bullying. Some of these previous investigations focused on children and/or adolescents (Führ, 2010; Proyer et al., 2012, 2013) and reported that higher gelotophobia was related to feelings of being a victim of bullying. Therefore, the importance of gelotophobia in school therapy practice has been already put forward (Bledsoe and Baskin, 2014; Platt et al., 2016). Considering workplace bullying – although the role of gelotophobia in workplace bullying has been theoretically discussed (e.g., Hofmann et al., 2017) – to our knowledge there are the only three empirical studies to date.

In a sample of adults, Platt (2008) found that participants who reported that were victims of bullying had higher gelotophobia scores (in comparison to individuals who did not disclose such an experience). It should be noted that it was not considered whether being a victim of bullying was specifically in the workplace. Gelotophobes [for cut-off points indicating slight pronounced, and extreme expression of gelotophobia, see Ruch and Proyer (2008b)] also did not discriminate between scenarios of ridicule (a form of bullying) and friendly teasing; individuals with extreme gelotophobia had same emotional reactions (i.e., disgust, surprise, and shame) to both types of interactions. Platt et al. (2009) confirmed and extended these findings, in particular by showing that being a victim of bullying was best predicted by high gelotophobia scores and by low happiness scores concerning playful teasing situations. Although some of the participants were recruited from an anti-workplace bullying support network group, the victim of bullying status was more broadly defined as in the previous study. The most recent panel study by Ruch and Stahlmann (2020) focused, among other things, on the relationship between gelotophobia and workplace bullying (workplace bullying was operationalized with the four-item Workplace Incivility Scale; Cortina et al., 2001), and found that there was a positive correlation between gelotophobia and workplace bullying in all of the six measurement intervals (waves) in their research.

Finally, it should be stressed that previous investigations have related gelotophobia to personality dimensions (Ruch et al., 2008, 2013; Hřebíčková et al., 2009; Ruch and Proyer, 2009; Proyer and Ruch, 2010; Ďurka and Ruch, 2015). Utilizing a Czech sample, Hřebíčková et al. (2009) reported that gelotophobia was associated with higher neuroticism, and lower extraversion, agreeableness, and openness to experience (neuroticism and extraversion showed the most robust relations); and concluded that personality dimensions play a significant role in whether individuals cope with ridicule easily, or whether they find it difficult. In general, gelotophobes can be described as introverted neurotics with a lower inclination to openness (Ruch et al., 2013). While personality characteristics have been studied in relation to gelotophobia, there is no previous research taking into account the joint consideration of personality and gelotophobia, and their relation to workplace bullying.

### Aim of the present study

The current study aimed to investigate the relationship between personality dimensions, gelotophobia, and exposure to workplace bullying. Furthermore, our specific aim was to explore whether gelotophobia has an incremental validity in predicting exposure to workplace bullying beyond the Big Five dimensions of personality. We expect that gelotophobia will have an incremental effect over and above personality dimensions in predicting exposure to workplace bullying (and especially humor-related bullying).

Two research gaps are considered in this study. First, the joint investigation of the Big Five dimensions and gelotophobia – and their relation to workplace bullying – has not been undertaken yet. Secondly, previous studies (relating gelotophobia with bullying) have either focused on children and/or adolescents (Führ, 2010; Proyer et al., 2012, 2013) or used only self-labeled victimization from bullying (Platt, 2008; Platt et al., 2009; cf., Ruch and Stahlmann, 2020). In general, we aim to extend previous findings by including a well-established behavioral type measure of workplace bullying (the Negative Acts Questionnaire-Revised – NAQ-R; Einarsen et al., 2009) and consider a humor-related bullying dimension (a specific sub-factor of person-related bullying) as well.

## Materials and methods

### Participants and procedure

Data was gathered online using software for online assessment (MindMap Diagnostic Methods). On the first page of the study link, participants were informed about the purpose of the study and it was stated that by continuing to fill in the questionnaires they provide an informed consent. In addition, it was stated that they should participate in the research if they are more than 6 months employed. The data collection was conducted from January 2023 to June 2023, and participants were recruited by social media posts on Facebook and LinkedIn. No monetary incentives were offered for participation. An email from one of the authors of this study was provided in case of further questions regarding the research. However, no participant utilized this option.

The study sample size was *a priori* estimated with power analysis on previous correlations of gelotophobia and workplace bullying (from Ruch and Stahlmann, 2020), where the mean zero-order correlation was *r* = 0.145. Using 85% power, a standard *α* = 0.05, and a one-sided test, the needed total sample would be 339 participants (performed with package pwr, Champely et al., 2020). We maximized our collection possibilities; in total, 482 participants opened the test battery, with 137 not completing all of the questionnaires. Therefore, only 345 individuals completed the test battery. The final sample consisted of 328 participants (77% females) as 17 participants were flagged as outliers (and removed from the dataset) using the Mahalanobis Distance procedure in the careless package [v1.1.3; Yentes and Wilhelm, 2018; see also Meade and Craig (2012) for identifying careless responding by using Mahalanobis Distances]. Data cleaning was conducted on the answers provided on the separate scales on the Big Five Inventory-2 (BFI-2-S; see Instruments section). Participants were from the Czech Republic and their age was between 20 and 66 years (*M* = 37.95, *SD* = 9.66). The sociodemographic characteristics of the sample are provided in the Supplementary Table S1.

### Instruments

#### Workplace bullying

Exposure to workplace bullying was assessed with the Czech version (Cakirpaloglu et al., 2017) of the NAQ-R (Einarsen et al., 2009). The questionnaire is comprised of 22 behavioral items measuring three factors (work-related bullying, person-related bullying, and physically intimidating bullying). For the purpose of this study, a humor-related bullying factor comprised of three items involving behaviors related to humor was calculated. The included items were “Being humiliated or ridiculed in connection with your work” (item 2); “Practical jokes carried out by people you do not get along with” (item 15) and, “Being the subject of excessive teasing and sarcasm” (item 20). The NAQ-R also includes self-labeled victimization from bullying during the last 6 months assessed with a single-item measure (item 23: Have you ever been bullied at work?) following a definition of workplace bullying (Einarsen and Skogstad, 1996; see also Supplementary Table S3). Participants choose one of the five alternatives on the behavioral items (“Never,” “Now and then,” “Monthly,” “Weekly,” and “Daily”) and the single-item measure [“no,” “yes, sometimes (rarely),” “yes, several times per month,” “yes, several times per week” and “yes, almost daily”]. Internal consistency in the present sample was high for all of the bullying dimensions as well as the total NAQ-R score, namely McDonald’s Omegas were 0.86 (work-related bullying), 0.95 (person-related bullying), 0.78 (physically intimidating bullying), 0.79 (humor-related bullying), and 0.96 (NAQ-R total score).

#### Personality domains

To assess the Big Five personality domains, we used the short form of the Big Five Inventory-2 (BFI-2-S) (Soto and John, 2017b; Hřebíčková et al., 2020). The scale is composed of 30 items, which have a common item stem (“I am someone who…”) and short descriptive phrases (e.g., “Tends to be quiet,” “Is temperamental, gets emotional easily”). A 5-point scale (ranging from disagree strongly to agree strongly) is utilized for the rating of each item by the participants. In this study, McDonald’s Omegas were 0.74 (extraversion), 0.74 (agreeableness), 0.77 (conscientiousness), 0.84 (negative emotionality), and 0.74 (open-mindedness).

#### Gelotophobia

Gelotophobia was measured using the Czech language version of the GELOPH<15> (Ruch and Proyer, 2008a; Hřebíčková et al., 2009), which is the psychometrically valid 15-item Czech language self-report instrument used for assessment of gelotophobia (e.g., “When others make joking remarks about me I feel being paralyzed”). Participants answer each item on a four-point scale (1 = “strongly disagree,” 4 = “strongly agree”). McDonald’s Omega was 0.93.

## Results

### Descriptive statistics

The descriptive statistics (mean, standard deviation, skewness, and kurtosis) of the measures are depicted in Table 1.

**Table 1:** Descriptive statistics of the measures used.

	*M*	*SD*	Skewness	Kurtosis
Extraversion	3.24	0.69	−0.13	−0.44
Agreeableness	3.81	0.60	−0.44	−0.13
Conscientiousness	3.76	0.65	−0.44	−0.05
Negative emotionality	2.90	0.78	0.25	−0.34
Open-mindedness	3.65	0.67	−0.06	−0.42
Gelotophobia	1.95	0.60	0.64	0.05
Work-related	1.89	0.81	1.49	2.06
Person-related	1.66	0.83	1.83	3.04
Physical intimidation	1.40	0.67	2.87	9.54
Humor-related	1.49	0.77	2.30	5.85
NAQ-R	1.70	0.74	1.76	3.03

*N* = 328.

Table 1 shows that skewness and kurtosis indicated substantial non-normality for the NAQ-R total score and the bullying dimensions (i.e., values for the skewness and/or kurtosis were greater than +2). For the rest of the measures, the values of the skewness and kurtosis could be considered acceptable [see Hair et al. (2022)].

Considering the prevalence of workplace bullying, in our sample it was found that 27.1% of the participants were exposed to workplace bullying when Leymann’s criterion (Leymann, 1996) was applied (i.e., facing at least one of the 22 negative acts on a weekly/daily basis during a minimum of 6 months). When a more strict criterion was used, or two acts on a week/daily basis (Mikkelsen and Einarsen, 2001), 12% of the participants could be classified as victims of workplace bullying. The prevalence of self-reported bullying was 9%. The percentage of endorsed behavioral items and the self-labeled victimization from bullying are given in the Supplementary Tables S2, S3.

### Intercorrelations

Pearson product–moment correlations between the Big Five domains, gelotophobia and exposure to workplace bullying are given in Table 2.

**Table 2:** Pearson product–moment correlations between the study variables.

	1	2	3	4	5	6	7	8	9	10	11
(1) Extraversion	—										
(2) Agreeableness	0.11^*^	—									
(3) Conscientiousness	0.35^***^	0.24^***^	—								
(4) Negative emotionality	−0.48^***^	−0.25^***^	−0.34^***^	—							
(5) Open-mindedness	0.17^**^	0.24^***^	0.15^**^	−0.07	—						
(6) Gelotophobia	−0.47^***^	−0.27^***^	−0.26^***^	0.54^***^	−0.10	—					
(7) Work-related	−0.05	−0.03	0.04	0.13^*^	0.11	0.25^***^	—				
(8) Person-related	−0.09	−0.06	0.00	0.15^**^	0.05	0.33^***^	0.79^***^	—			
(9) Physically intimidating	−0.07	−0.06	0.02	0.13^*^	−0.02	0.27^***^	0.59^***^	0.75^***^	—		
(10) Humor-related	−0.13^*^	−0.04	−0.02	0.19^***^	0.01	0.36^***^	0.67^***^	0.90^***^	0.72^***^	—	
(11) NAQ-R	−0.08	−0.05	0.02	0.15^**^	0.06	0.32^***^	0.90^***^	0.97^***^	0.78^***^	0.87^***^	—
(12) Self-labeled victimization	−0.05	−0.01	0.03	0.10	0.05	0.20^***^	0.64^***^	0.77^***^	0.57^***^	0.70^***^	0.76^***^

*N* = 328. ^*^p < 0.05, ^**^p < 0.01, ^***^p < 0.001.

Table 2 shows that negative emotionality was positively related to the NAQ-R total score, and to each of the bullying dimensions, as expected. However, there was not a statistically significant relation between negative emotionality and self-labeled victimization from bullying. The rest of the Big Five traits were not related to any of the bullying measures, with an exception of the significant negative association between extraversion and humor-related bullying. In line with the expectations, gelotophobia correlated positively with the NAQ-R total score, the separate bullying dimensions, and the self-labeled victimization from bullying. The relation between personality and gelotophobia also corroborated previous findings, as gelotophobia was related to each of the personality domains, and the most robust correlations were found with extraversion and negative emotionality. Finally, self-reported victimization from bullying was strongly related to the NAQ-R total score and its dimensions.

In order to correct for the substantial non-normality of the exposure to bullying measures, we also calculated Spearman rank order correlations (see Supplementary Table S4). Each of the above mentioned findings were replicated (except for the extraversion relation to humor-related bullying, which cease to be significant), albeit the correlations were generally weaker.

### Regression analysis

We conducted two hierarchical multiple regression models and used the NAQ-R total score as a dependent variable. Model 1 included the personality domains (Big Five), whereas Model 2 incorporated both personality domains and gelotophobia to assess its incremental effect on bullying. A summary of both models can be found in Table 3. Assumptions of linear regression were mostly met, however residuals had a minor deviation from normal distribution.

**Table 3:** Linear regression models for exposure to workplace bullying (NAQ-R total score).

	Variable	*b*	95% CI for *B*		SE *b*	Beta	$R^{2}$	$R_{adj.}^{2}$	$\Delta R^{2}$
			LL	UL					
Model 1							0.04^*^	0.02^*^	
	(Intercept)	0.92	−0.08	1.92	0.51				
	Open-mindedness	0.09	−0.03	0.21	0.06	0.08			
	Negative emotionality	0.15*	0.03	0.27	0.06	0.16			
	Conscientiousness	0.10	−0.04	0.23	0.07	0.09			
	Extraversion	−0.04	−0.18	0.09	0.07	−0.04			
	Agreeableness	−0.06	−0.20	0.08	0.07	−0.05			
Model 2							0.12^**^	0.10^**^	0.09
	(Intercept)	−0.2	−1.23	0.84	0.53				
	Open-mindedness	0.09	−0.03	0.21	0.06	0.08			
	Negative emotionality	0.02	−0.10	0.15	0.06	0.02			
	Conscientiousness	0.10	−0.03	0.23	0.07	0.09			
	Extraversion	0.07	−0.07	0.20	0.07	0.06			
	Agreeableness	0.01	−0.13	0.15	0.07	0.01			
	Gelotophobia	0.46^**^	0.30	0.62	0.08	0.37			

*N* = 328. CI, confidence interval; LL, lower limit; UL, upper limit. ^*^p < 0.05, ^**^p < 0.01.

In the first model, negative emotionality was the only statistically significant predictor of the NAQ-R total score; beta = 0.16 [95% CI (0.03, 0.29), *t*(322) = 2.43, *p* = 0.016]. However, in the second model, gelotophobia had the only significant effect [beta = 0.37, 95% CI (0.24, 0.50), *t*(321) = 5.61, *p* < 0.001], and the difference in $R^{2}$ between Models 1 and 2 is $\Delta R^{2}$ = 0.086.

Next, humor-related bullying was used as a dependent variable. Similar results occurred (see Table 4), where only gelotophobia was statistically significant in the second model [beta = 0.39, 95% CI (0.26, 0.52), *t*(321) = 5.98, *p* < 0.001].

**Table 4:** Linear regression models for exposure to humor-related bullying.

	Variable	*b*	95% CI for *b*		SE *b*	Beta	$R^{2}$	$R_{adj.}^{2}$	$\Delta R^{2}$
			LL	UL					
Model 1							0.04^*^	0.02^*^	
	(Intercept)	0.88	−0.15	1.91	0.52				
	Open-mindedness	0.03	−0.10	0.15	0.07	0.02			
	Negative emotionality	0.18^**^	0.05	0.31	0.06	0.18			
	Conscientiousness	0.07	−0.07	0.21	0.07	0.06			
	Extraversion	−0.07	−0.21	0.07	0.07	−0.06			
	Agreeableness	−0.01	−0.16	0.14	0.07	−0.01			
Model 2							0.14^**^	0.12	0.96
	(Intercept)	−0.34	−1.40	0.72	0.54				
	Open-mindedness	0.02	−0.10	0.14	0.06	0.02			
	Negative emotionality	0.04	−0.09	0.17	0.07	0.04			
	Conscientiousness	0.07	−0.06	0.20	0.07	0.06			
	Extraversion	0.05	−0.09	0.19	0.07	0.04			
	Agreeableness	0.06	−0.08	0.21	0.07	0.05			
	Gelotophobia	0.50^**^	0.33	0.66	0.08	0.39			

*N* = 328. CI, confidence interval; LL, lower limit; UL, upper limit. ^*^p < 0.05, ^**^p < 0.01.

## Discussion

The current study aimed to explore and advance knowledge pertaining to the relation between personality dimensions, gelotophobia, and exposure to workplace bullying. The specific goal was to investigate the incremental validity of gelotophobia in predicting workplace bullying beyond the personality dimensions. We firstly focus on the prevalence of workplace bullying in our sample, and afterwards discuss the main findings of our study.

The percentage of individuals who were classified as victims of workplace bullying (using both the looser and stricter criterion) via the behavioral items are comparable to previous studies. For example, the study by Cakirpaloglu et al. (2017), which was also done in a Czech context utilizing a sample of 7,103 employees, reported that 24.78% (using the looser criterion) and 14.84% (using the stricter criterion) of the participants in their study could be classified as victims of bullying. Furthermore, we have also found a drop of the percentage when the self-labeling approach was applied, and the percentage of individuals who self-labeled as victims of bullying in our study is similar to previous investigations (e.g., Einarsen et al., 2009; Tsuno et al., 2010; Dujo López et al., 2020).

The correlational findings indicated that negative emotionality was related to exposure to workplace bullying (i.e., experienced negative acts). This is in line with the meta-analytic investigation by Nielsen et al. (2017) which concluded that neuroticism is the strongest and most consistent correlate of exposure to harassment. It should be noted that each of the three mechanisms indicating an association between individual dispositions and bullying (Nielsen and Knardahl, 2015) can be applied to explain this relation [see Nielsen et al. (2017); see also Djurkovic et al. (2006) and Bowling et al. (2010)]. The present results confirmed previous investigations and extend to a different cultural context. Moreover, we have utilized a more recent operationalization of the neuroticism (i.e., negative emotionality) construct – which was not considered in Nielsen et al. (2017) – comprised of the facets of anxiety, depression, and emotional volatility (Soto and John, 2017b).

It is interesting to note that we have not confirmed the results considering the relation between the other personality dimensions (i.e., extraversion, agreeableness, and conscientiousness) and workplace bullying. However, this can be explained by the fact that one of the moderators for this association was the geographical region [see Nielsen et al. (2017)]. In particular, the relation between agreeableness and conscientiousness, and exposure to workplace harassment was moderated by the geographical region, that is higher estimates were found in studies from the United States compared to Europe (for agreeableness), and studies from the USA and Asia/Oceania compared to Europe (for conscientiousness). As the Czech Republic represents a central European context, our findings confirm this trend. However, the non-significant association between extraversion and workplace bullying cannot be explained by the moderator analyses reported by Nielsen et al. (2017), and needs further consideration.

Gelotophobia can be placed on introverted neurotic personality dimensions with a lower inclination to openness (Ruch et al., 2013). While personality characteristics have been studied in relation to gelotophobia, this is the first study that accounts for the joint consideration of personality and gelotophobia, and their relation to workplace bullying. Focusing specifically on the gelotophobia and exposure to workplace bullying relation, the results indicated that gelotophobia was positively related to both the behavioral measures of bullying and the self-labeled victimization. Furthermore, it was a stronger correlate to workplace bullying than neuroticism, and in line with theoretical considerations, the most robust relation was found with humor-related bullying. In general, the association between gelotophobia and workplace bullying can also be theoretically explained using the mechanisms proposed by Nielsen and Knardahl (2015), as analogous to the personality dimensions – bullying relation.

Gelotophobia overlaps with neuroticism/negative emotionality in regard to behaviors related to nervousness and insecurity (e.g., indicators of gelotophobia are difficulty to hold eye contact and stiffness) and these behaviors could be seen by others as annoying. Thus, making gelotophobes both provocative and easy targets of bullying. This is consistent with the explanation applying the target behavior mechanism for the neuroticism–harassment relation (Milam et al., 2009; Nielsen et al., 2017).

Next, following the negative perception mechanism, individuals with high gelotophobia scores might have a lowered threshold for interpreting behaviors as harassing/bullying, especially behaviors related to humor. Moreover, the perceived bullying could constitute a “false alarm,” or in reality, there is a lack of objective proof for it (Platt et al., 2009; Ruch, 2009; Hofmann et al., 2017). In other words, gelotophobes might misinterpret the good-humored teasing from their colleagues, superiors, or subordinates, as being ridiculed/bullied (Platt, 2008).

Finally, workplace bullying might in fact be an antecedent of gelotophobia, as proposed by the reverse causality mechanism. This especially could be true in workplaces with extreme humor culture (Plester, 2016; Plester et al., 2022) and/or negative humor climate (Cann et al., 2014), and is in accordance with the proposed causes of gelotophobia. According to Ruch et al. (2014), in adulthood, intense traumatic experiences of being laughed at or ridiculed is one of the causes of gelotophobia. Therefore, individuals can become gelotophobic as a consequence of working in an extreme ‘fun culture.’

### The incremental validity of gelotophobia

To the best of our knowledge, our study is the first to provide evidence for the incremental validity of gelotophobia beyond the personality domains. Although negative emotionality and gelotophobia overlap to a certain degree, the results from the regression analysis indicated that gelotophobia predicted exposure to workplace bullying (operationalized with the total score on the NAQ-R) over and above negative emotionality. More interestingly, gelotophobia was the only significant predictor of humor-related bullying, which is in line with theoretical considerations (e.g., Hofmann et al., 2017). In sum, we provide initial evidence that the construct of gelotophobia should be considered, over and above the personality dimensions, in order to understand exposure to workplace bullying.

## Limitations and future directions

Besides the general limitations common to cross-sectional design studies, and utilizing convenience sampling, there are also some further limitations to our study. First, we have not focused on investigating whether there are different homogenous groups, which may vary based on the nature and extent of their exposure to bullying (Notelaers et al., 2006; Einarsen et al., 2009). Therefore, future studies might employ latent class cluster analysis [e.g., Magidson and Vermunt, 2004; see also Reknes et al. (2021)] and also investigate how the identified groups differ regarding the personality dimensions and gelotophobia. Second, recent methodological considerations propose that incremental validity should be tested using structural equation modeling (SEM) (Wang and Eastwick, 2020; Feng and Hancock, 2022). For example, Wang and Eastwick (2020) warn that standard multiple regression inflates Type 1 error. Therefore, our results need to be replicated using SEM. It should be stressed that such research would require large sample sizes; possibly in the thousands, in order to reach a desirable level of power (Wang and Eastwick, 2020). Large sample sizes are also required if one is utilizing the latent class cluster approach [see Nylund-Gibson and Choi (2018)].

Furthermore, we did not explore the contribution of the facets of personality dimensions, and future investigations could focus on the relation between specific facets of personality and their relation to exposure to workplace bullying. Moreover, we used the short version of the BFI-2, while future research should employ the full version (Soto and John, 2017a); especially when the interest is on the facets of personality. Research is needed that will focus particularly on the humor-related bullying and its relation to gelotophobia. Platt (2021) expressed the need to include samples which include greater levels of the higher ranges on the distribution of gelotophobia on the continuum of fear – from no fear to extreme fear of being laughed at. This would be an important consideration of further studies, given the reported prevalence of gelotophobia in Hřebíčková’s et al. (2009) study that indicated the low percentage of gelotophobes in the Czech Republic [e.g., slight (5.24 %) marked (1.05 %)] and no reported extreme gelotophobes being identified [for comparable countries see Platt and Forabosco (2012)]. As Platt (2021) reports, it is only at the marked and extreme levels that strong pathological effects of gelotophobia are observed. These investigations should also consider the humor culture/climate in the specific organizations, which could include qualitative focus groups statements that would identify any mis-interpretation of pro-social humorous interaction as bullying.

## Conclusion

Bullying behavior is detrimental for the bully victim. This is further impacted on in a workplace which can have serious financial as well as emotional consequences. This study has supported the link between workplace bullying and gelotophobia from the framework of personality and providing initial incremental validity for this relationship.

## Data availability statement

The raw data supporting the conclusions of this article will be made available by the authors, without undue reservation.

## Ethics statement

Ethical approval was not required for the studies involving humans because the study adhered to ethical principles outlined by APA. The studies were conducted in accordance with the local legislation and institutional requirements. The participants provided their written informed consent to participate in this study.

## Author contributions

FS: Conceptualization, Data curation, Formal analysis, Methodology, Writing – original draft, Writing – review & editing. KP: Data curation, Formal analysis, Methodology, Writing – original draft. JG: Conceptualization, Data curation, Writing – original draft. TP: Writing – original draft, Writing – review & editing. MS: Data curation, Writing – review & editing.
