# Supplementary material for: Exposure to workplace bullying: the incremental effect of gelotophobia beyond the big five
Source: Front Psychol. 2024 Apr 25;15:1400940. doi: 10.3389/fpsyg.2024.1400940 (PMC11079308; doi:10.3389/fpsyg.2024.1400940)
Supplement: Supplementary file 1 [file Data_Sheet_1.docx]

Supplementary Material

# Supplementary Tables

**Supplemental Table S1.** Sociodemographic characteristics of the participants.

|  | *n* | % |
| --- | --- | --- |
| **Sex** |  |  |
| Male | 77 | 23 |
| Female | 251 | 77 |
| **Education level** |  |  |
| Elementary | 3 | 1 |
| Graduated without matriculation | 20 | 6 |
| Secondary school with matriculation | 109 | 33 |
| Higher vocational education | 13 | 4 |
| Higher education up to Bachelor level | 35 | 11 |
| Higher education | 148 | 45 |
| **Employment sector** |  |  |
| Public | 101 | 31 |
| Non-profit | 22 | 7 |
| Private | 205 | 63 |
| **Years of employment in the particular sector** |  |  |
| Up to one | 26 | 8 |
| 1 – 4,99 | 97 | 30 |
| 5 – 9,99 | 72 | 22 |
| 10 and more | 133 | 41 |
| **Region of living in the Czech republic** |  |  |
| Prague | 78 | 24 |
| Central Bohemian | 44 | 13 |
| South Bohemian | 7 | 2 |
| Plzeň | 8 | 2 |
| Karlovy Vary | 3 | 1 |
| Ústí nad Labem | 32 | 10 |
| Liberec | 0 | 0 |
| Hradec Králové | 6 | 2 |
| Pardubice | 6 | 2 |
| Vysočina | 15 | 5 |
| South Moravian | 88 | 27 |
| Olomouc | 15 | 5 |
| Zlín | 22 | 7 |
| Moravian-Silesian | 4 | 1 |

**Supplemental Table S2.** Percentage of endorsed behavioral items on the NAQ-R.

|  |  | *Never (%)* | *Now and*  *then (%)* | | *Monthly (%)* | *Weekly (%)* | *Daily (%)* |
| --- | --- | --- | --- | --- | --- | --- | --- |
| 1 | Someone withholding information  which affects your performance | 34 | 45 | 6 | | 9 | 6 |
| 2 | Being humiliated and ridiculed in  connection with your work | 55 | 27 | 3 | | 9 | 5 |
| 3 | Being ordered to do work below your level of competence | 38 | 47 | 5 | | 6 | 4 |
| 4 | Having key areas of responsibility  removed or replaced with more trivial or unpleasant task | 54 | 31 | 5 | | 6 | 3 |
| 5 | Spreading of gossips and rumours about you | 33 | 45 | 5 | | 8 | 9 |
| 6 | Being ignored or excluded | 53 | 31 | 4 | | 8 | 5 |
| 7 | Having insulting and offensive remarks made about your attitudes or your private life | 57 | 28 | 1 | | 9 | 4 |
| 8 | Being shouted at or being the target of spontaneous anger | 52 | 35 | 5 | | 4 | 4 |
| 9 | Intimidating behaviour such as finger pointing, invasion of personal space, shoving, blocking your ways | 78 | 15 | 2 | | 2 | 3 |
| 10 | Hint or signals from others that you  should quit the job | 77 | 16 | 2 | | 2 | 2 |
| 11 | Repeated reminders of your errors and mistakes | 54 | 32 | 5 | | 5 | 4 |
| 12 | Being ignored or facing a hostile  reaction when you approach | 69 | 20 | 3 | | 3 | 5 |
| 13 | Persistent criticism of your errors and mistakes | 63 | 21 | 5 | | 5 | 5 |
| 14 | Having your opinion ignored | 40 | 43 | 4 | | 7 | 7 |
| 15 | Practical jokes carried out by people you do not get along with | 84 | 13 | 1 | | 1 | 1 |
| 16 | Being given task with unreasonable  deadlines | 50 | 36 | 5 | | 5 | 4 |
| 17 | Having allegations made against you | 60 | 26 | 5 | | 6 | 4 |
| 18 | Excessive monitoring of your work | 52 | 30 | 4 | | 6 | 6 |
| 19 | Pressure not to claim something to  which by right you are entitled (e.g., sick leave, holiday, entitlement) | 63 | 25 | 5 | | 3 | 3 |
| 20 | Being the subject of excessive teasing and sarcasm | 75 | 16 | 2 | | 3 | 3 |
| 21 | Being exposed to an unmanageable  workload | 39 | 39 | 7 | | 7 | 8 |
| 22 | Threats of violence or physical abuse or actual abuse | 95 | 3 | 1 | | 1 | 1 |

**Supplemental Table S3.** Percentage of endorsement on the single-item measure of workplace bullying (item 23)

| *Have you ever been bullied at work? Bullying takes place when one or more persons systematically and over time feel that they have been subjected to negative treatment on the part of one or more persons, in a situation in which the person(s) exposed to the treatment*  *have difficulty in defending themselves against them. We do not consider a single incident to be workplace bullying*.  *Using this definition, please indicate whether you have been subjected to bullying in the workplace in the last 6 months.* | |
| --- | --- |
| *Responses* | *Frequency (%)* |
| No | 71 |
| Yes, sometimes (rarely) | 13 |
| Yes, several times per month | 8 |
| Yes, several times per week | 4 |
| Yes, almost daily | 5 |

**Supplemental Table S4.** Spearman rank order correlations between the study variables.

|  | 1 | 2 | 3 | 4 | 5 | 6 | 7 | 8 | 9 | 10 | 11 |
| --- | --- | --- | --- | --- | --- | --- | --- | --- | --- | --- | --- |
| (1) Extraversion | — |  |  |  |  |  |  |  |  |  |  |
| (2) Agreeableness | .10 | — |  |  |  |  |  |  |  |  |  |
| (3) Conscientiousness | .32*** | .23*** | — |  |  |  |  |  |  |  |  |
| (4) Negative emotionality | -.46*** | -.25*** | -.30*** | — |  |  |  |  |  |  |  |
| (5) Open-mindedness | .18*** | .22*** | .13* | -.08 | — |  |  |  |  |  |  |
| (6) GELOT | -.44*** | -.28 | -.26*** | .49*** | -.11* | — |  |  |  |  |  |
| (7) Work-related | -.02 | -.05 | .03 | .14** | .09 | .22*** | — |  |  |  |  |
| (8) Person-related | -.00 | -.03 | .03 | .15** | .06 | .20*** | .78*** | — |  |  |  |
| (9) Physically intimidating | .01 | .01 | .03 | .12* | -.01 | .11* | .58*** | .64*** | — |  |  |
| (10) Humor-related | -.05 | -.03 | .03 | .18*** | .04 | .22*** | .67*** | .85*** | .59*** | — |  |
| (11) NAQ-R | -.01 | -.05 | .03 | .16** | .08 | .22*** | .92*** | .95*** | .70*** | .82*** | — |
| (12) Self-labelled victimization | -.01 | -.02 | .02 | .10 | .05 | .13* | .57*** | .69*** | .51*** | .65*** | .68*** |

*Note*. *N* = 328.

* p < .05, ** p < .01, *** p < .001.
